# A randomised trial of [^18^F]PSMA‐1007‐PET/CT versus NaF‐PET/CT for staging primary prostate cancer: A trial protocol

**DOI:** 10.1002/bco2.243

**Published:** 2023-05-12

**Authors:** Karen Middelbo Buch‐Olsen, Mads Hvid Poulsen, Steinbjørn Hansen, Mie Holm Vilstrup, Jorun Holm, Søren Hess, Paw Christian Holdgaard, Karsten Egbert Arnold Zieger, Søren Sørensen Madsen, Oke Gerke, Kasper Tholstrup Pedersen, Johan Hygum Dam, Niels Langkjær, Louise Dorner Østergaard, Jon Thor Asmussen, Poul Erik Braad, Birgitte Nørgaard, Matthias Eiber, Malene Grubbe Hildebrandt

**Affiliations:** ^1^ Department of Nuclear Medicine Odense University Hospital Odense C Denmark; ^2^ MANTRA ‐ Centre for MagNetic resonance Technology for Response Adapted radiotherapy Odense University Hospital Odense Denmark; ^3^ Department of Clinical Research University of Southern Denmark Odense Denmark; ^4^ Department of Urology OUH Odense Odense C Denmark; ^5^ Department of Urology The Hospital of South West Jutland Esbjerg Denmark; ^6^ Department of Oncology OUH Odense Odense C Denmark; ^7^ Department of Radiology and Nuclear Medicine The Hospital of South West Jutland Esbjerg Denmark; ^8^ PREMIO ‐ Centre for Personalized Response Monitoring in Oncology Odense University Hospital Odense Denmark; ^9^ IRIS ‐ Imaging Research Initiative Southwest Hospital South West Denmark Esbjerg Denmark; ^10^ Department of Regional Health Research, Faculty of Health Sciences University of Southern Denmark Odense Denmark; ^11^ Department of Nuclear Medicine Lillebaelt University Hospital of Southern Denmark Vejle Denmark; ^12^ Department of Urology Lillebaelt University Hospital of Southern Denmark Vejle Denmark; ^13^ Department of Radiology OUH Odense Odense C Denmark; ^14^ Department of Clinical Engineering Region of Southern Denmark Vejle Denmark; ^15^ Department of Public Health University of Southern Denmark Odense C Denmark; ^16^ Department of Nuclear Medicine Technical University of Munich Munich Germany; ^17^ Centre for Innovative Medical Technology Odense University Hospital Odense Denmark

**Keywords:** primary prostate cancer, progression‐free survival, PSMA‐PET/CT, quality of life, staging

## Abstract

**Background:**

Prostate‐specific membrane antigen (PSMA)‐positron emission tomography/contrast‐enhanced computed tomography (PET/CT) is a sensitive imaging modality for prostate cancer (PCa). Due to lack of knowledge of the patient benefit, PSMA‐PET/CT is not yet recommended in the European guidelines for staging and treatment planning of patients with newly diagnosed PCa. We will investigate the potential difference in progression‐free survival (PFS) and quality of life (QoL) of using PSMA‐PET/CT versus sodium fluoride (NaF)‐PET/CT for staging and treatment planning in patients with newly diagnosed PCa.

**Study Design:**

This is a prospective randomised controlled multicentre trial carried out at three centres in the Region of Southern Denmark.

**Endpoints:**

The primary endpoint is PFS. Secondary endpoints are residual disease, stage migration, impact on treatment strategies, stage distribution, QoL and diagnostic accuracy measures.

**Patients and Methods:**

Patients eligible for the study have newly diagnosed unfavourable intermediate‐ or high‐risk PCa. A total of 448 patients will be randomised 1:1 into two groups: (A) a control group staged with Na[^18^F]F‐PET/CT and (B) an intervention group staged with [^18^F]PSMA‐1007‐PET/CT. A subgroup in the intervention group will have a supplementary blinded Na[^18^F]F‐PET/CT performed for the purpose of performing accuracy analyses. QoL will be assessed at baseline and with regular intervals (3–12 months) during the study period. Treatment decisions are achieved at multidisciplinary team conferences based on the results of the respective scans and according to current Danish guidelines.

**Trial Registration:**

The Regional Committees on Health Research Ethics for Southern Denmark (S‐20190161) and the Danish Medicines Agency (EudraCT Number 2021‐000123‐12) approved the study, and it has been registered on clinicaltrials.gov (Record 2020110469).

## 1 BACKGROUND

Prostate cancer (PCa) is one of the most frequent cancer forms in men in developed countries with 1.4 million patients diagnosed in 2020 and approximately 375 000 deaths worldwide due to PCa the same year.^1^ The 5‐year relative survival rate approaches 100% for the vast majority (89%) of men diagnosed with PCa but drops to 30% for those diagnosed with stage IV disease.^2^

For primary staging, current guidelines recommend traditional imaging, including bone scintigraphy (BS) and contrast‐enhanced computed tomography (CECT). These are, however, rather inaccurate with reported sensitivities and specificities of 42%–80% and 82%, respectively.^3^, ^4^ BS reflects bone metabolism and may be replaced by the newer Na[^18^F]F‐positron emission tomography (PET) although this has not yet proven any added value.^5^

The transmembrane protein, prostate‐specific membrane antigen (PSMA), is expressed on the majority of PCa cells and has become a unique target for molecular imaging of PCa. PSMA ligands can be labelled with positron‐emitting isotopes such as ^18^F and ^68^Ga for imaging. The ^18^F isotope is beneficial regarding availability and production. PSMA‐1007 is only sparsely excreted in the urinary tract, making diagnostics in the pelvic region favourable.^6^

Accuracy studies of PSMA‐PET/CT for lymph node staging in the primary setting have reported moderate sensitivities (59%) and high specificities (93%), but most studies have been performed with a ^68^Ga‐labelled tracer.^7^, ^8^ It has recently been shown in a randomised prospective trial that [^68^Ga]Ga‐PSMA‐11 PET/CT had a 27% greater accuracy compared with conventional imaging with CECT and BS. Upstaging and management change were reported 22% and 13% more frequently for PSMA‐PET/CT compared with conventional imaging, respectively.^9^ Regarding the favourable [^18^F]PSMA‐1007‐PET/CT, a multicentre retrospective study has shown promising sensitivity and specificity for lymph node staging of 85.9% and 99.5%, respectively.^10^

The European guidelines report that it could be tempting to replace BS and CECT with PSMA‐PET/CT in initial PCa staging, but the clinical benefit of detecting metastases at an earlier time point is still undetermined. Therefore, it is argued that results from randomised controlled trials (RCTs) evaluating the management and outcome should be available before a decision can be made to treat patients based on the results of these tests.^11^ We are aware of neither previous studies, current studies nor studies being planned to evaluate the impact of replacing traditional imaging with up‐to‐date PSMA‐PET/CT on patient outcomes such as progression‐free survival (PFS) and quality of life (QoL).

This study aims to provide knowledge of the role of PSMA‐PET/CT in the primary staging of PCa to improve evidence‐based clinical decision‐making.

### 1.1 Objectives

The overall aim of this project is to investigate the impact on PFS and QoL of using [^18^F]PSMA‐1007‐PET/CT for primary staging in PCa compared with Na[^18^F]F‐PET/CT. We will compare [^18^F]PSMA‐1007‐PET/CT (interventional imaging) with Na[^18^F]F‐PET/CT (conventional imaging at our centres) in a randomised controlled design with regard to PFS (primary endpoint), stage distribution, treatment strategy, frequency of residual disease and QoL (secondary endpoints). We aim to analyse the diagnostic accuracy, stage migration and change in treatment strategy for [^18^F]PSMA‐1007‐PET/CT compared with Na[^18^F]F‐PET/CT in a subgroup.

### 1.2 Study design

**FIGURE 1:**
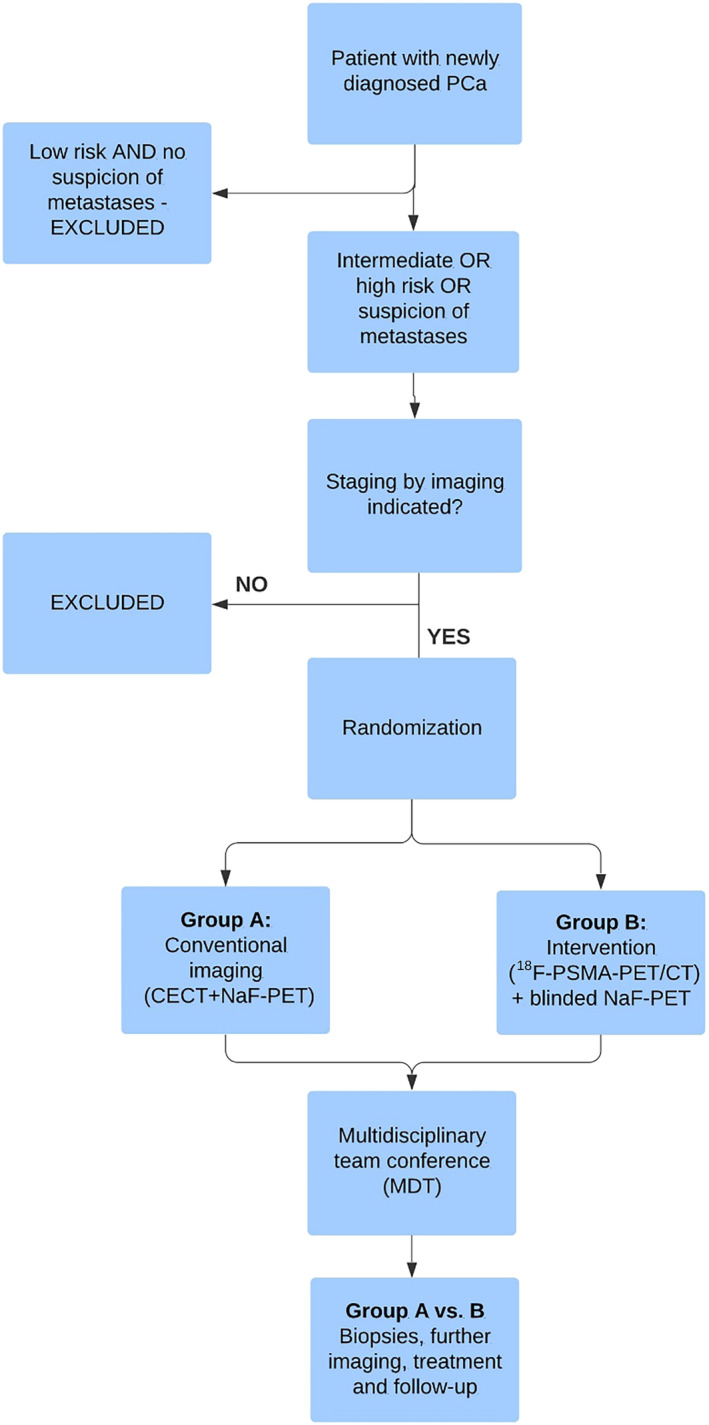
Flow chart for PRISMA‐positron emission tomography (PET). CECT, contrast‐enhanced computed tomography; CT, computed tomography; PCa, prostate cancer; PSMA, prostate‐specific membrane antigen.

This trial is a randomised controlled clinical trial following the Consolidated Standards of Reporting Trials (CONSORT) guidelines.^12^ A total of 448 patients (power calculation is in the statistical section) will be included and randomised into a control group (A) and an intervention group (B) (Figure 1 and Appendix A). Patients in Group A will be staged by Na[^18^F]F‐PET/CT, that is, CECT for lymph node and soft tissue metastases, and Na[^18^F]F‐PET for bone metastases according to current practice at our centres. Patients in Group B will be staged by [^18^F]PSMA‐1007‐PET/CT (including CECT) for lymph node, bone and soft tissue metastases. A subgroup of patients in Group B will have a blinded Na[^18^F]F‐PET scan with low‐dose CT (ldCT) for attenuation correction for the purpose of performing comparative accuracy analyses.

### 1.3 Eligibility criteria

The study population comprises men (aged 18 years or older) with newly diagnosed unfavourable intermediate‐ or high‐risk (including locally advanced) PCa^13^ or low‐risk PCa with clinical suspicion of metastases based on other findings (i.e., bone pain or suspicion of lymph node or bone metastasis on the magnetic resonance imaging [MRI] performed before prostate biopsies). The inclusion and exclusion criteria are listed in Table 1. Patients will be recruited from the three urologic departments in the Region of Southern Denmark and referred for imaging prior to treatment in the clinical set‐up. Scans will be performed in three nuclear medicine departments in the region.

**TABLE 1:** Inclusion and exclusion criteria for patient enrolment.

Inclusion criteria, all must be fulfilled	Exclusion criteria, any criterion fulfilled warrants exclusion
Has given informed consent to participate	Consent not given
Can read and understand provided patient information material in Danish	Inability to read and/or understand provided patient information material in Danish
Biopsy verified, newly diagnosed and untreated prostate cancer OR Biopsy performed because of clinically strong suspicion of prostate cancer	Previously given consent to this study withdrawn for any reason
Any, some or all of the following features: PSA ≥ 20 ng/mL OR Gleason score ≥ 4 + 3 OR Tumour stage cT2c or above as determined by digital rectal exploration and/or transrectal ultrasonography Suspicion of metastases as judged clinically, for example, bone pain	Staging by imaging not warranted as judged clinically
Staging by imaging is warranted	Allergy towards Na[^18^F]F, PSMA or other contents in the solutions
	Biopsy performed because of clinically strong suspicion of prostate cancer not confirming the diagnosis
	PSA value above 200 ng/mL

Abbreviations: cT2c, clinical stage T2c; PSA, prostate‐specific antigen; PSMA, prostate‐specific membrane antigen.

## 2 METHODS

### 2.1 Enrolment and randomisation

Patient recruitment will be carried out in a collaborative effort by the regional departments of urology and nuclear medicine. Patients will receive oral and written study information, and acceptance to participate is documented in signed consent forms. Consenting patients will be randomised and allocated into one of the study groups. Randomisation will be performed 1:1 into Groups A and B with stratification according to risk status of high, unfavourable intermediate and low risk with suspected metastases, respectively. The randomisation will be executed in the REDCap database software (https://projectredcap.org/).

### 2.2 PET radiotracers

The [^18^F]PSMA‐1007 will be produced according to Good Manufacturing Practice (GMP) in laboratory facilities at the hosting nuclear medicine department at Odense University Hospital. The [^18^F]PSMA‐1007 will be distributed to the two collaborating nuclear medicine departments, Hospital Lillebaelt and Hospital South West Jutland. The radiopharmaceutical will be dosed 2 MBq/kg, minimum 150 MBq and maximum 300 MBq. Sodium fluoride (Na[^18^F]F) will be partly produced at the hosting nuclear medicine department at Odense University Hospital and partly in a different facility in Denmark (Skejby Hospital). For the Na[^18^F]F‐PET, we will use a fixed dose of 200 MBq. See Table 2 for tracer specifications.

**TABLE 2:** Radiopharmaceutical specifications.

	Specification	
Radiopharmaceutical	[^18^F]F‐PSMA‐1007	Na[^18^F]F
Radioactivity, total (GBq)	≤40.9	≤123.7
Product volume (mL)	18 ± 1	17 ± 0.5
Appearance	Clear and colourless (slightly yellow)	Clear and colourless (slightly yellow)
pH	6.5–8.5	5.0–8.5
Chemical purity		NA
Impurities, single (mg)	≤0.1	
Impurities, total (mg)	≤0.5	
Radiochemical purity (%)	≥95	≥98.5
Radionuclidic purity (%)	≥99.9	≥99.9
Sterility	No growth	No growth
Endotoxins (IU/mL)	<9.7	<10.3
[^18^F]F‐PSMA‐1007, mass (mg)	≤1.8 mg	NA
Residual solvents		NA
Ethanol (%)	≤10	
Dimethylsulfoxide (%)	<0.25	
Content of		NA
Tetrabutylammonium ion (mg)	≤2.6	
Sodium ascorbate (mg)	≤105	
Half‐life, *T*½ (min)	105–115	105–115
γ‐Spectrometry (KeV)	511	511
Shelf‐life (h)	5	10

Abbreviations: NA, not applicable; PSMA, prostate‐specific membrane antigen.

### 2.3 Imaging techniques

The PET/CT scanners are state‐of‐the art scanners from GE (GE Discovery 710 and GE Discovery MI, Hospital South West Jutland; GE Discovery MI, Odense University Hospital) and Siemens (Siemens Biograph mCT Flow 64 and Siemens Biograph Vision 600 Edge, Hospital Lillebaelt). All patients will have a CECT (according to local guidelines for contrast administration) as performed clinically in these patients in each department. The [^18^F]PSMA‐1007‐PET/CT scans will be performed 60 min post injection of the tracer, and Na[^18^F]F‐PET/CT 30 min post tracer injection.

### 2.4 Image interpretation

Experienced nuclear medicine physicians and radiologists will read the examinations, and the [^18^F]PSMA‐1007‐PET/CT scans will be evaluated according to Prostate Cancer Molecular Imaging Standardized Evaluation (PROMISE),^14^ where lesions in lymph nodes, bones and other tissues are categorised into five categories ranging from benign to malignant (Appendix B).

The Na[^18^F]F‐PET/CT scans will be evaluated according to a similar Likert scale for bone lesions. On the CECT, the lymph nodes in the pelvic region will be considered possibly malignant if more than 8 mm in short axis, round and with no fat in the hilum.^11^

### 2.5 Reference standard

Metastatic disease will be deemed without further imaging or examinations if the presentation is considered of typical morphology and pattern for PCa, especially considering lymph nodes and bones. In other tissues, metastases will also be suspected by a typical appearance, and biopsy will be intended only as a part of the clinical set‐up when possible and clinically relevant. Biopsies are not performed solely as a part of the study. Further imaging will be used as reference standard to confirm or exclude metastatic disease when clinically indicated (see Table 3).

**TABLE 3:** Reference standard.

	Metastases deemed
Any organ but the prostate gland	Pathology confirming metastasis
Lymph nodes	Equal to or more than 8 mm, round and with no fat in the hilum, for PSMA‐PET with uptake of PSMA
Bones	Marked uptake of activity with or without malignant morphology in CT and not related to joint with degenerative changes or a fracture, or malignant morphology with uptake
Liver	Biopsy if possible and clinically relevant, for PSMA‐PET with uptake of PSMA
Lung nodules—malignant	Biopsy if possible (and clinically relevant)
Disseminated disease	If disseminated disease with a typical pattern for prostate cancer, disseminated disease can be determined without further imaging or biopsy
Metastases	Further imaging supporting the suspicion of a metastasis

Abbreviations: CT, computed tomography; PET, positron emission tomography; PSMA, prostate‐specific membrane antigen.

### 2.6 Clinical management

The patients will be discussed in a multidisciplinary team (MDT) conference based on the results of the scans and staging performed accordingly. If in doubt of metastases, further imaging or biopsy will be performed if possible and relevant. The MDT will suggest a treatment strategy that follows the contemporary national guidelines.^15^ In a shared decision process with the patient, the final choice of treatment will be decided upon, and the patients may be treated by prostatectomy, radiotherapy, androgen deprivation therapy (ADT), chemotherapy or antiandrogens and, eventually, in combinations.

### 2.7 QoL

QoL will be assessed using the following widely used questionnaires—all of them were translated and cross‐culturally validated in Danish:

- Functional Assessment of Cancer Therapy‐Prostate (FACT‐P)—39 items^16^;
- European Quality of Life‐Five Dimensions (EQ‐5D‐5L) covering five dimensions: mobility, self‐care, usual activities, pain/discomfort and anxiety/depression—25 items and a VAS scale^17^, ^18^, ^19^; and
- The Expanded Prostate Cancer Index Composite (EPIC‐26)—26 items.^20^

The patients will be asked to fill in the questionnaires electronically or on paper at fixed time points in their pathway (at diagnosis and after 3, 6, 12, 24, 60, 120 and 234 months after enrolling—enabling data collection for approximately 20 years after inclusion of the last patient). Time points of evaluation are chosen to be able to evaluate QoL over time after diagnosis and treatment.

## 3 PRIMARY AND SECONDARY ENDPOINTS

The primary endpoint, PFS, will be compared between Groups A and B and defined as the time from inclusion to progression (Appendix C) or death for any reason. Progression is defined as clinical or biochemical recurrence/progression. Progression can be considered with or without progression on imaging or change in treatment. After prostatectomy, a confirmed rise of PSA (two following rising PSA values according to Prostate Cancer Working Group 3 [PCWG3]^21^, ^22^) above 0.2 ng/mL for patients with unmeasurable PSA value after prostatectomy will deem progression. For patients after radiotherapy, a confirmed rise in PSA value above 2 ng/mL from nadir defines recurrence. Patients with measurable PSA after prostatectomy and subsequent referral to salvage radiotherapy for completion of treatment will not be categorised as having progression but as having residual disease (Appendix D). Residual disease will be a secondary endpoint compared between the two groups too.

Progression is determined for patients undergoing palliative treatment with a subsequent confirmed rising PSA and/or progression on imaging, leading to initiation or change of treatment.^23^

The distribution of disease stage and treatment strategy will be evaluated between the two groups as secondary endpoints. Overall survival is a secondary endpoint too and will be evaluated between Groups A and B after suitable time spans, expected after 5, 10, 15 and 20 years. Regarding QoL, changes from baseline to 12 months are of primary interest, and an 8‐point decline in the global FACT‐P score will be considered clinically relevant. We suggest that more sensitive and suggestively correct staging will lead to improved QoL, because the treatment initiated is expected to better fit each patient. For example, small metastases that might not be identified with Na[^18^F]F‐PET/CT can be taken into account and leave the patients with less unattended small metastatic lesions and hence avoiding initial curative intended treatment with possible side effects for patients with primary disseminated disease.

### 3.1 Diagnostic accuracy and stage migration

The secondary endpoint of diagnostic accuracy and stage migration will be performed in a subgroup of patients within Group B where patients will act as their own controls. Hence, the comparison will be made for Na[^18^F]F‐PET/CT versus [^18^F]PSMA‐1007‐PET/CT for diagnosing bone, lymph node and soft tissue metastases. For this purpose, the CECT from the [^18^F]PSMA‐1007‐PET/CT will be used together with the blinded Na[^18^F]F‐PET scan, which will be performed with a ldCT for sparing radiation to participating patients. In the same subgroup, we will determine the rate of stage migration and change of treatment strategy. This will be performed in an MDT‐similar set‐up for research purposes only. In this MDT, clinical information about the patients and the blinded Na[^18^F]F‐PET/CT scans will be used to make a first decision on the treatment strategy, which will subsequently be compared with the clinically decided treatment strategy based on the [^18^F]PSMA‐1007‐PET/CT. Due to potential stage migration in the PSMA group, we expect a change of treatment strategy in a smaller group of patients.

### 3.2 Statistical analysis plan and sample size determination

Descriptive statistics will be done according to data type: mean ± standard deviation or median (range) for symmetrically and asymmetrically distributed continuous variables, respectively, as judged visually with histograms including approximating normal curves; and frequencies and percentages for categorical variables.

The analysis of time‐to‐event endpoints (PFS, overall survival) will comprise Cox proportional hazard regressions adjusted for group, risk status (high‐, intermediate‐ and low‐risk PCa) and centre. Graphical displays will comprise Kaplan–Meier plots including risk tables (i.e., patients at risk in each group) and the results of respective log‐rank tests. The analysis of QoL data will comprise the estimation of the group difference in a repeated measurement analysis. Descriptive analyses will be performed, and results presented by proportions. Continuous variables will be compared using a *t* test, and categorical variables using a chi‐squared test. Additionally, regression analysis will be used to identify associations within data. Accuracy assessments of Na[^18^F]F‐PET/CT and [^18^F]PSMA‐1007‐PET/CT will relate to sensitivity, specificity, positive and negative predictive values that will be judged upon the reference standard (Table 3). Exploratory, bivariate testing will consist of unpaired *t* tests, alternatively, Wilcoxon's rank sum test, on continuous variables and chi‐squared test, alternatively, Fisher's exact test, on categorical variables. The level of significance will be 5% (two‐sided). All analyses will be done with STATA/IC 17 (StataCorp, College Station, TX 77845, USA).

A total of 402 patients (201 in each arm) are sufficient to indicate a statistically significant difference in PFS with assumed hazard rates of 0.35 and 0.5 for [^18^F]PSMA‐1007‐PET/CT and conventional staging, respectively, implying a hazard ratio of 0.7. Estimates are based on a study duration of 4 years, consisting of 3 years of accrual time and a minimum follow‐up period of 1 year (two‐sample comparison of survivor functions; exponential test; power: 80%). Accounting for loss to follow‐up of 10%, a total of 448 patients (224 in each arm) will be included in the study.

Regarding the subgroup analysis of diagnostic accuracy of [^18^F]PSMA‐1007‐PET/CT compared with Na[^18^F]F‐PET/CT, we assumed a prevalence of metastases of 30%, sensitivity and specificity of 0.38 and 0.91 for conventional imaging, respectively, and 0.85 and 0.98 for [^18^F]PSMA‐1007‐PET/CT, respectively. Then, a sample size of 128 patients is sufficient to indicate a superior sensitivity for [^18^F]PSMA‐1007‐PET/CT and a non‐inferior specificity for [^18^F]PSMA‐1007‐PET/CT with a non‐inferiority margin of 0.052 (power: 80%). This subgroup analysis will be made and published when the first 128 patients have been recruited in Group B.

### 3.3 Interim analysis

We assume the distribution of patients across stages N_0_M_0_, N_0_M_1A_, N_0_M_1B_, N_1_M_0_, N_1_M_1A_ and N_1_M_1B_, to differ between Na[^18^F]F‐PET/CT and [^18^F]PSMA‐1007‐PET/CT (for a mock example, see Appendix E). More specifically, we assume the sum of *absolute* differences in the proportions of patients in the different stages to differ by at least 20%.^10^, ^24^ In the mock example (Appendix E), this value is 30%. A difference of at least 20% is a necessary, but not a sufficient condition for an advantage of [^18^F]PSMA‐1007‐PET/CT over Na[^18^F]F‐PET/CT with respect to the primary endpoint as both treatment choice and treatment effect will influence PFS in both study arms.

One interim analysis will be performed on the first *N* = 270 included patients, aiming for 60% of the overall recruitment target of 448 patients. The purpose of this interim analysis will be a comparison of the distribution of patients across stages N_0_M_0_, N_0_M_1A_, N_0_M_1B_, N_1_M_0_, N_1_M_1A_ and N_1_M_1B_ by study arm as described. This assessment will be done internally by KMB‐O and OG. If this difference (Na[^18^F]F‐PET/CT minus [^18^F]PSMA‐1007‐PET/CT) falls short of 15%, stopping the study early due to futility will be considered by the study core team, consisting of KMB‐O, SH, OG and MGH.

This interim analysis enables a potential early stopping due to futility, not due to early success. The primary hypothesis will *not* be tested in the interim analysis.

## 4 DISCUSSION

Several studies have shown that PSMA‐PET/CT is superior to BS and CECT for detecting relapse of PCa, and PSMA‐PET/CT is introduced in the guidelines for this indication.^24^, ^25^ The first studies on PSMA‐PET/CT for primary staging of PCa have been published, showing higher sensitivity to detecting metastases than BS and CECT.^26^, ^27^ PSMA‐PET/CT is only cautiously introduced in the international guidelines for primary staging of intermediate‐ and high‐risk PCa patients because of lack of evidence of whether the increased sensitivity will translate into patient benefit of being staged with this more sensitive modality.^11^

Hofman et al. have performed a large randomised controlled study in a cross‐over design.^9^ They randomised the patients in two groups: One group was initially staged with conventional imaging (BS and CECT) and another group with [^68^Ga]Ga‐PSMA‐11 PET/CT. If less than three metastases were found in either group at initial staging, the patients crossed over to the other group and a new staging was performed. Follow‐up was performed clinically and with imaging in up to 2 years after the recruitment of the last patient. They found that [^68^Ga]Ga‐PSMA‐11 PET/CT had higher accuracy than conventional imaging, and a change of treatment strategy was seen in 27% of patients initiating with conventional imaging, whereas 5% changed treatment strategy after crossing over to conventional imaging. However, the study was not designed to provide information on patient benefit of using PSMA‐PET/CT as frequently requested, for example, in the international guidelines.^11^ Information such as the gain in PFS, residual disease after treatment with curative intent and QoL is still lacking.

In this study, we hypothesise that staging patients with PSMA‐PET/CT will lead to a longer PFS. Today, a significant group of PCa patients are diagnosed with residual disease after prostatectomy.^28^, ^29^ We believe that more sensitive imaging for primary staging will reduce this rate because of more correct initial staging and improved treatment for the individual patient, and ultimately a longer PFS, a longer overall survival, and improved QoL.

We expect stage migration in a group of patients staged with [^18^F]PSMA‐1007‐PET/CT, where small lymph node metastases may be found in the pelvis or along the abdominal aorta—metastases that would not have been detected with CECT because of their small size. These patients will be presented at MDT conferences and, here, the conclusion may end up with a change from the standard treatment (with curative intent) to an individualised treatment towards both the prostate gland and the lymph node metastases locally or distant; hence, the treatment may include surgery as well as radiation and ADT. Because of possible stage migration in the PSMA group, we do not expect that the treatment proportions will balance between the two arms. Our observation from current clinical practice is that some patients staged with Na[^18^F]F‐PET/CT experience residual disease or recurrence short time after initial treatment with curative intent. These patients are currently being referred to a [^18^F]PSMA‐1007‐PET/CT to detect residual disease or metastases.

The recommendations for treatment have changed over the last decade^11^ and with current opportunities, even localised treatment of a solitary metastasis may be considered relevant.^30^ This means that using sensitive procedures for primary staging should be considered even more important than ever before.

The strengths of this study are the prospective design in a multicentre setting and with clinically and patient‐relevant outcomes. We have replaced conventional BS with Na[^18^F]F‐PET/CT clinically in the participating centres in this project, which may be considered a limitation because the majority of clinical trials have used BS for treatment planning. However, as Na[^18^F]F‐PET/CT is not proven more sensitive than BS and CECT, we believe that the results will still be relevant for centres using BS and CECT for staging.

The fact that we expect some patients in the PSMA arm to be up‐staged, a higher selection of patients in the PSMA group will be offered treatment with curative intent. This will affect the comparability of the groups with respect to defining PFS, which is a limitation to the study. The different treatment options offered to the patients over a long period of time after the diagnosis could be considered a limitation too, as well as the different definitions of progression according to the different treatments.

## 5 CONCLUSION

There is a need for prospective randomised multicentre studies with patient‐related outcome and long‐term evaluation of using PSMA‐PET/CT for primary staging of unfavourable intermediate‐ and high‐risk PCa patients. We find that this trial is clinically important for evaluating the usefulness of PSMA‐PET/CT in primary staging of PCa before being implemented as a standard clinical procedure for this patient group.

## AUTHOR CONTRIBUTIONS

*Study concept and design*: Karen Middelbo Buch‐Olsen, Mads Hvid Poulsen, Steinbjørn Hansen, Mie Holm Vilstrup, Oke Gerke, Kasper Tholstrup Pedersen and Malene Grubbe Hildebrandt. *Drafting of the manuscript*: Karen Middelbo Buch‐Olsen and Malene Grubbe Hildebrandt. *Critical revision of the manuscript for important intellectual content*: All authors. *Statistical analysis*: Karen Middelbo Buch‐Olsen and Oke Gerke. *Obtained funding*: Karen Middelbo Buch‐Olsen and Malene Grubbe Hildebrandt. *Study supervision*: Matthias Eiber, Oke Gerke and Malene Grubbe Hildebrandt.

## CONFLICT OF INTEREST STATEMENT

Karen Middelbo Buch‐Olsen, Mads Hvid Poulsen, Steinbjørn Hansen, Mie Holm Vilstrup, Jorun Holm, Søren Hess, Paw Christian Holdgaard, Søren Sørensen Madsen, Oke Gerke, Kasper Tholstrup Pedersen, Johan Hygum Dam, Niels Langkjær, Louise Dorner Østergaard, Jon Thor Asmussen, Poul Erik Braad, Birgitte Nørgaard and Malene Grubbe Hildebrandt declare no conflicts of interest or financial ties to disclose. Matthias Eiber declares receipt of grants from Blue Earth Diagnostics; consulting fees from Blue Earth Diagnostics Ltd, Novartis/AAA, Point Biopharma, Telix, Rayzebio and Janssen Pharmaceuticals; payment for lectures/presentations/manuscript writing/educational events from Janssen Pharmaceuticals; payment for expert testimony from Paraxel and Bioclinica; support for attending meetings/travel from Blue Earth Diagnostics and Terumo; patents planned, issued or pending for rbPSMA; leadership/board committee for EANM Committee Meeting; and stock or stock options from Novartis, Telix, Bayer and Point Biopharma. Karsten Egbert Arnold Zieger declares receipt of grants from Photocure A/S (research support for bladder cancer); receipt of consulting fee from Photocure A/S; and support for attending meeting/travel from Bayer A/S (NUF 2022 Helsinki).

## Appendix B INTERPRETATION CRITERIA USED FOR THE [^18^F]PSMA‐1007‐PET/CT ACCORDING TO PROMISE^15^

**Table:**

**1**	Definitively benign/no evidence of disease
**2**	Tracer uptake in sites atypical for metastases—likely benign/unlikely malignant
**3**	May be benign or malignant—require further workup?
**4**	Lesions with high tracer uptake typical for PCa, but lack of anatomy/suggestive of malignancy
**5**	High tracer uptake and corresponding anatomy, definitively cancer/consistent with malignancy

Abbreviations: PCa, prostate cancer; PROMISE, Prostate Cancer Molecular Imaging Standardized Evaluation.

## Appendix C PRIMARY AND SECONDARY STUDY ENDPOINTS

**Table:**

**Primary endpoint**	
Group A versus Group B	Progression‐free survival
**Secondary endpoints**	
Group A versus Group B	Stage distribution; Treatment distribution; Frequency of residual disease; QoL; Overall survival
**Secondary endpoint**	
Na[^18^F]F versus PSMA in Group B	Accuracy of evaluating bone metastases; Stage migration; Treatment strategy

Abbreviations: PSMA, prostate‐specific membrane antigen; QoL, quality of life.

## Appendix D DEFINITIONS OF RECURRENCE AND PROGRESSION^15^

**Table:**

**Recurrence after prostatectomy**	PSA rising from unmeasurable to above 0.2 ng/mL in two following measurements.
**Recurrence after radiotherapy**	PSA rising with 2 ng/mL above nadir in two following measurements.
**Progression**	PSA rising in two following measurements AND/OR progression on imaging AND/OR clinical progression.
**Castration resistance**	Progression despite of castration.

Abbreviation: PSA, prostate‐specific antigen.

## Appendix E MOCK DISTRIBUTION OF NM STAGES BY ARM

**Table:**

Stage	Na[^18^F]F‐PET/CT	[^18^F]PSMA‐1007‐PET/CT	Difference
N_0_M_0_	55%	45%	+10%
N_0_M_1A_	14%	13%	+1%
N_0_M_1B_	14%	14%	0%
N_1_M_0_	13%	24%	−11%
N_1_M_1A_	4%	0%	+4%
N_1_M_1B_	0%	4%	−4%

Abbreviations: CT, computed tomography; PET, positron emission tomography; PSMA, prostate‐specific membrane antigen.
